# Discrepancy between significant fibrosis and active inflammation in patients with cardiac sarcoidosis: combined and image fusion analysis of cardiac magnetic resonance and ^18^F fluorodeoxyglucose positron emission tomography

**DOI:** 10.1186/s41824-019-0056-4

**Published:** 2019-06-14

**Authors:** Kenji Fukushima, Michinobu Nagao, Atsushi Yamamoto, Naoki Serizawa, Umiko Ishizaki, Atsushi Suzuki, Akiko Sakai, Eri Watanabe, Mitsuru Momose, Ichiei Kuji, Koichiro Abe

**Affiliations:** 1 Department of Nuclear medicine, and Cardiovascular Center, Saitama Medical University International Center, Yamane1397-1, Hidaka City, Saitama Japan; 2 Department of Diagnostic imaging and Nuclear medicine, Tokyo Women’s Medical University, Kawada-cho 8-1, Shinjuku, Tokyo, Japan; 3 Department of Cardiology, Tokyo Women’s Medical University, Kawada-cho 8-1, Shinjuku, Tokyo, Japan

**Keywords:** Cardiac sarcoidosis, Cardiac MRI, FDG-PET, Image fusion

## Abstract

**Background:**

Diagnosis and evaluation of cardiac sarcoidosis (CS) are mainly based on the combined use of cardiac magnetic resonance imaging (CMR) and ^18^F fludeoxyglucose positron emission tomography (FDG). Though these modalities can detect the pathological feature of the disease, combined assessment has not been fully examined. Multimodality image fusion is known to be useful for further comprehension, while most image interpretation is performed with a side by side comparison in clinical routine. We investigated the similarity and discrepancy of active inflammation, regional fibrosis, and wall function by image fusion of CMR and FDG.

**Methods:**

Patients with CS who underwent both CMR and FDG were retrospectively enrolled. The extent of myocardial late gadolinium enhancement (LGE) in left ventricle (LGE volume), cardiac function, and volume (left ventricular ejection fraction, LVEF; end-diastolic volume, EDV) was measured from CMR. The FDG uptake in whole myocardium (whole SUVmax), cardiac metabolic volume (CMV), and cardiac metabolic activity (CMA) was calculated from FDG. CMR and FDG were fused and divided into AHA 17 model for segmental analysis. Wall motion, the magnitude of LGE in myocardial wall (LGE%wall), and corresponding FDG uptake (segmental SUVmax) were analyzed.

**Results:**

Forty-one patients were retrospectively enrolled. In patients with FDG uptake, LVEF inversely correlated to LGE volume and positively correlated to SUVmax (*r* = − 0.56, *p* < 0.0001, and *r* = 0.08, *p* = 0.048, respectively). Discrepancy between LGE volume and CMV showed a significant positive correlation to whole SUVmax and CMA (*r* = 0.49, *p* < 0.0001, and *r* = 0.96, *p* < 0.0001, respectively). In image fusion analysis, segmental SUVmax showed a significant inverse correlation to LGE%wall (Spearman’s rank correlation coefficient; *r* = − 0.15, *p* = 0.008). LGE%wall also showed significant inverse correlation to wall motion (*r* = − 0.13, *p* = 0.0011).

**Conclusion:**

Combined and fusion analysis with CMR and FDG demonstrated the discrepancy of myocardial inflammation and extensive fibrosis. Active inflammation was present in the earlier stage of myocardial fibrosis and was found to be less in the wall with advanced fibrosis and remodeling. Combined analysis of CMR and FDG can incrementally reclassify the pathological stage of CS.

## Introduction

Cardiac sarcoidosis is a disease of granulomatous inflammation which leads to the patient’s adverse outcome (Birnie et al. 2016). Unlike ischemic heart disease and the other cardiomyopathy, anti-inflammatory therapy is first-line therapy (Kusano and Satomi 2016; Yazaki et al. 2001). Hence, precise diagnosis and detection of myocardial inflammation are pivotal to identify the appropriate candidates. However, reliable diagnosis is not fully established, and the latest guidelines from Japan and Europe propose diagnosis by noninvasive multi-modal imaging for clinical diagnosis (Hiraga et al. 2007; Birnie et al. 2014). Currently, cardiac magnetic resonance imaging (CMR) and ^18^F fludeoxyglucose positron emission tomography (FDG) are widely used for the detection of myocardial fibrosis and active inflammation. However, FDG findings may vary according to the presence and the activity of myocardial inflammation and disease progression among each individual, and late gadolinium enhancement (LGE) findings are not capable of identifying the appropriate candidate for anti-inflammatory therapy (Vita et al. 2018). To date, the relation of magnitude of fibrosis and active inflammation was rarely investigated, while both have established quantitative measurement technique to classify the progression or activity of the disease. Standard deviation method is a common technique to measure LGE volume in left ventricle. In FDG, cardiac metabolic volume and cardiac metabolic activity that are originally proposed in the field of oncology (i.e., metabolic tumor volume, total lesion glycolysis) are also available using a certain threshold. Notably, combined and quantitative analysis of myocardial fibrosis and active inflammation has been rarely reported, and only a few studies reported to evaluate active inflammation and therapeutic effect using a certain threshold (Osborne et al. 2014; Ahmadian et al. 2014).

The purpose of this study was to compare and contrast the magnification of myocardial fibrosis, the impairment of wall motion, and the activity of inflammation in patients with cardiac sarcoidosis (CS) by image fusion analysis of CMR and FDG by patient-based and quantitative methods.

## Methods

### Patients

We retrospectively investigated consecutive patients with CS who underwent both CMR and FDG between October 2013 and May 2017. Patients were retrospectively diagnosed based on the latest guidelines by the Japanese Circulation Society in 2017 and the expert consensus recommendation on criteria by the Heart Rhythm Society in 2014 (Hiraga et al. 2007; Birnie et al. 2014; Terasaki et al. 2018). All patients underwent CMR at first, then FDG within 3 months because of positive LGE findings consistent with CS in the process of diagnosing non-ischemic cardiomyopathy. All patients were referred for coronary angiography with computed tomography or invasive coronary angiography and negative for significant coronary artery disease.

Exclusion criteria are the following: (1) Patients who have received anti-inflammatory therapy including corticosteroid in any case. (2) Patients with past history of other cardiac disease (coronary artery disease, ischemic cardiomyopathy, hypertrophic cardiomyopathy, hypertensive heart disease, acute or chronic myocarditis except CS, tachycardia induced, post status of cardiac or thoracic aortic operation, or significant valvular disease). (3) Failure in high-fat and low-carbohydrate diet at FDG scans. (4) Poor image quality, unable for image fusion, or contraindication for CMR. (5) According to the reports by Osborne and Ahmadian, patients without significant FDG uptake (whole myocardial SUVmax < 2.7) were excluded (Osborne et al. 2014; Ahmadian et al. 2014).

Clinical history was reviewed in medical chart or/and referral document from other local hospitals. The findings in electrocardiogram and ventricular arrhythmic events in Holter monitoring were corrected. Patients were defined to have systemic sarcodidosis when they have been with biopsy-proven extra-cardiac sarcoidosis or showed significant extra-cardiac uptake in FDG scans.

### CMR protocol

Electrocardiogram (ECG)-gated CMR was performed in breath-hold using 1.5T Ingenia or 3.0T Intera (Phillips Medical System, the Netherlands) with a four-element phased-array coil in a supine position with breath-holds during expiration and ECG gating as suggested by the Society for Cardiovascular Magnetic Resonance/ European Cardiovascular Magnetic Resonance recommendations (Kramer et al. 2008). Cine was performed using a conventional steady-state-free-precession sequence. Cine images were acquired along an atrioventricular groove from the basal edge to the apical for long and short-axis view images. Following were the imaging parameters: repetition time 2.8 ms, echo time 1.4 ms, flip angle 45°, slice thickness 8 mm, field of view 380 mm, matrix size 176 × 193, SENSE factor 2, and 20 cardiac phases/RR intervals of the ECG. LGE images were acquired 6–10 min after administration of contrast media (gadodiamide or gadopentetate dimeglumine; 0.15 mmol/kg) using segmented inversion recovery adjusting inversion time to optimally null myocardium. In the short axis, cine and LGE were acquired on the same oblique and prescribed every 10 mm (slice thickness 6 mm, resolution 1.2–1.8 mm) from base to apex. CMR images were reviewed by an experienced radiologist (M.N.) and cardiologist (A.S.). Typical localization and patterns of epicardial to transmural hyperenhancement were considered positive LGE finding for CS. In the long axis 4-chamber cine images, thickness of basal septum was visually accessed and thinned wall compared to other segment was defined as basal thinning in interventricular septum.

### ^18^F FDG-PET protocol

Patients were undertaken a high-fat and low-carbohydrate diet 24 h prior FDG scan and at least over 18 h of fasting based on the guideline of the Japanese Society of Nuclear Cardiology (Manabe et al. 2016). After confirmation of the diet and fasting in the interview, intravenous administration of heparin (50 unit/kg) was performed and followed by FDG (150–220 MBq) injection at an interval of 15 min.

FDG images were obtained on a 64-slice PET/CT scanner with time of flight (Bio mCT, Siemens healthcare, Erlangen, Germany). Ninety minutes after FDG injection, cardiac scan was performed. Acquisition was performed from the craniocaudal direction with a 2-min emission per bed position from the mid-thigh to the skull base with 120 kVp and 50 mAs. CT images acquired with respiratory gating or shallow-expiration breath-hold were used as a transmission map for attenuation correction. Two experienced nuclear medicine physicians (UI and MM) interpreted FDG images. FDG uptake was measured by MN oncology in Syngo.via® (Siemens healthcare). Volume of interest was drawn around the heart not to include the liver, stomach, and mediastinum. Whole myocardial FDG uptake (whole SUVmax) was measured. The volume of standardized uptake value (SUV) over 2.7 was measured as cardiac metabolic volume (CMV mL), and cardiac metabolic activity (CMA) was calculated by multiplying SUV volume and mean SUV according to previous reports (Osborne et al. 2014).

### Functional and segmental analysis in CMR

**Fig. 1:**
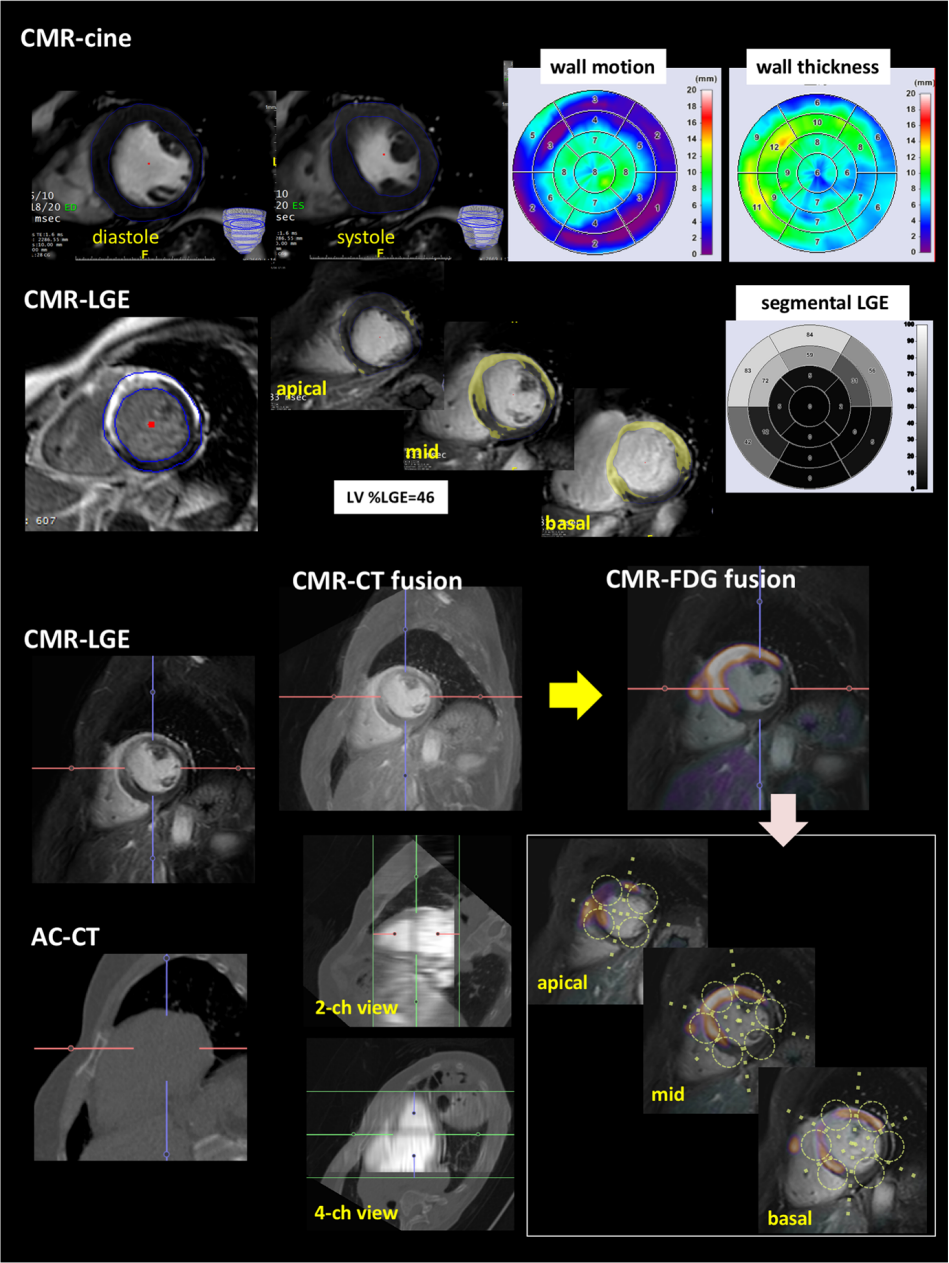
Schematic presentation of regional analysis by CMR-FDG image fusion was shown Fig. 1. In cine-MRI, myocardial contour was manually traced from apical to basal of the short axis slices, and allotted to short axis LGE image under same longitudinal axis. Short axis image for cine and LGE was converted to AHA 17 segment model. As the lower part of figure showed, short axis LGE dicom data and attenuation corrected CT (ACCT) were fused automatically by fusion tool (Intuition sever®). After confirmation of corresponding images or manual adjustment, ACCT was replaced by FDG-PET dicom data (yellow arrow). On basis of standardized AHA 17 segment model, the corresponding FDG uptake (segmental SUVmax) was measured by drawing volume of interest on PETCT under the guidance of CMR-FDG image fusion

The quantitative analysis of CMR was performed using commercially available software (Intuition server®. Terarecon co. ltd., Tokyo, Japan) (Fig. 1). In cine-MRI, epi- and end-myocardial contours in systolic and diastolic phase were defined for each short slice from basal to apex. Segmental wall motion and thickness were analyzed and converted to 17 AHA map by trisecting along longitudinal axis (Fig. 1 upper). Same axis definition was allotted to the short axis of LGE. The extent of LGE was quantified by the standard deviation (SD) methods. After null myocardium was defined as a remote reference, signal intensity thresholds over 6 SD were automatically measured. The extent of LGE in the whole left ventricle was calculated as LGE volume (cm^3^), and the percentage in LV was measured as LGE%LV, and segmental extent of LGE in myocardial wall was converted to AHA 17-segment map by trisecting LV for output as LGE%wall as well as cine-MRI (Fig. 1 upper middle) (Crawford et al. 2014).

### Patient-based segmental analysis by automatic CMR and FDG image fusion

The process of CMR and FDG image fusion is shown in the bottom part of Fig. 1. In CS, image fusion would be frequently unsuccessful either by automatic or manual due to the pathological nature of myocardial uptake (focal, multi-focal, or focal on diffuse). Thus, we performed image fusion by replacement technique using the volume fusion tool in Intuition server®. First, dicom data for CMR and attenuation correction CT (ACCT) of FDG were automatically fused, and the manual adjustment was done if necessary (Fig. 1 middle part of lower). Fused images were confirmed by observation on multi-planar reconstructed views (horizontal, vertical long, and short axis). Second, ACCT was replaced by an FDG image which was closely synchronized in 3D orientation with ACCT. Under the image fusion-guided process, LV was trisected along longitudinal axis from basal to apex as defined in cine and LGE, and volume of interest was drawn manually on each short axis of fused CMR-LGE and FDG images from apical to basal level according to 17 AHA model. Corresponding segmental SUVmax was measured on FDG under confirmation of co-registration of CMR and FDG.

### Data analysis

All data was expressed as mean ± SD. Linear regression and Spearman’s rank correlation were enrolled in correlation analysis in graphs. Statistical analysis was done by PRISM® ver.7 (Graph Pad Software, CA). The value of two-tailed *p* < 0.05 was considered significant.

## Results

### The patients

**Fig. 2:**
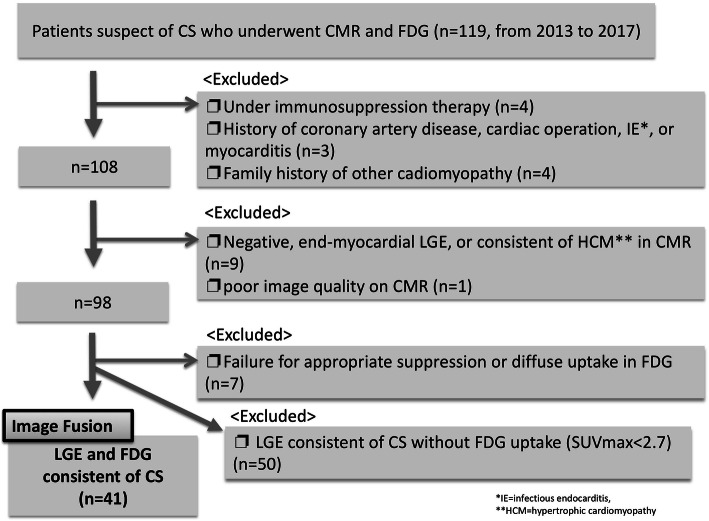
Flow chart of enrolled patients was shown. Out of total 119 patients who underwent CMR and FDG, patients were excluded with the following steps: (1) Under immunosuppression therapy, coronary artery disease, other inflammation, and other known cardiomyopathy; (2) LGE negative, CMR finding consistent with other cardiac diseases (i.e. Hypertrophic cardiomyopathy), and poor image; (3) failure of suppression of myocardial uptake or diffuse uptake; and (4) negative FDG uptake (whole SUVmax < 2.7). Finally, 41 patients were analyzed

Patient selection flow chart is shown in Fig. 2. As a result, 41 patients were enrolled into analysis. Four patients were under anti-inflammatory therapy, 3 had a history of coronary artery disease, and 4 had a significant family history of dilated cardiomyopathy. Nine showed LGE consistent of other cardiac disease, 7 patients failed in diet protocol for FDG, and 50 patients did not show significant myocardial FDG uptake (SUVmax < 2.7). Table 1 shows the profile of the enrolled patients. For we enrolled the patients according to the interpretation of CMR and FDG, all of them met the diagnostic criteria for clinical diagnosis by the Heart Rhythm Society (the “CMR protocol” section).

**Table 1:** Patient characteristics (total cohort = 41)

Age	62 ± 25.2
Female	60%
Asian	100%
ECG abnormality (AVB, BBB, PVCs*)	67%
Ventricular tachycardia	15%
Palpitation	54%
Syncope	24%
Heart failure	43%
JCS** 2017 criteria	
Major	
1 major	61%
2 major or over	26%
Minor	
0 minor	9%
1 minor	11%
2 minor	36%
3 minor or over	42%
HRS*** 2014 expert consensus criteria	
1 major	30%
2 major	100%
LVEF	34.2 ± 13.5%
LVEDV	133.2 ± 57.1 mL
LVESV	82.5 ± 54.0 mL
LGE volume	30.5 ± 26.4 mL
Whole myocardial SUVmax	8.7 ± 4.1
SUV volume	70.6 ± 101.8 mL

Data was described as mean value or percentage

*Atrioventiricular block, right or left bundle branch block, or premature ventricular contraction

**Japanese Circulation Society

***Heart Rhythm Society

### Cardiac function, LGE volume, and whole SUVmax

**Fig. 3:**
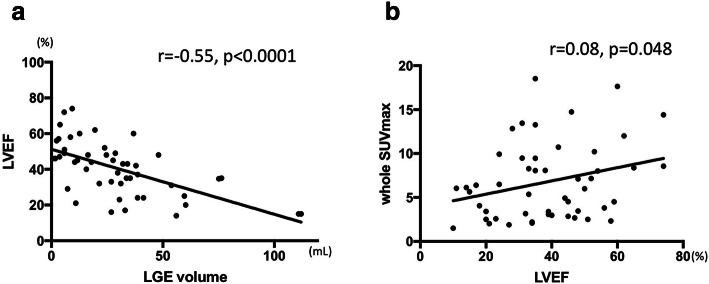
Patient-based analysis of LGE, cardiac function, volumetric, and FDG uptake (whole SUVmax) is shown. **a**, **b** The relationship between the severity of LGE in LV (%LV LGE) vs. LVEF and LVEDV which had significant correlation

**Fig. 4:**
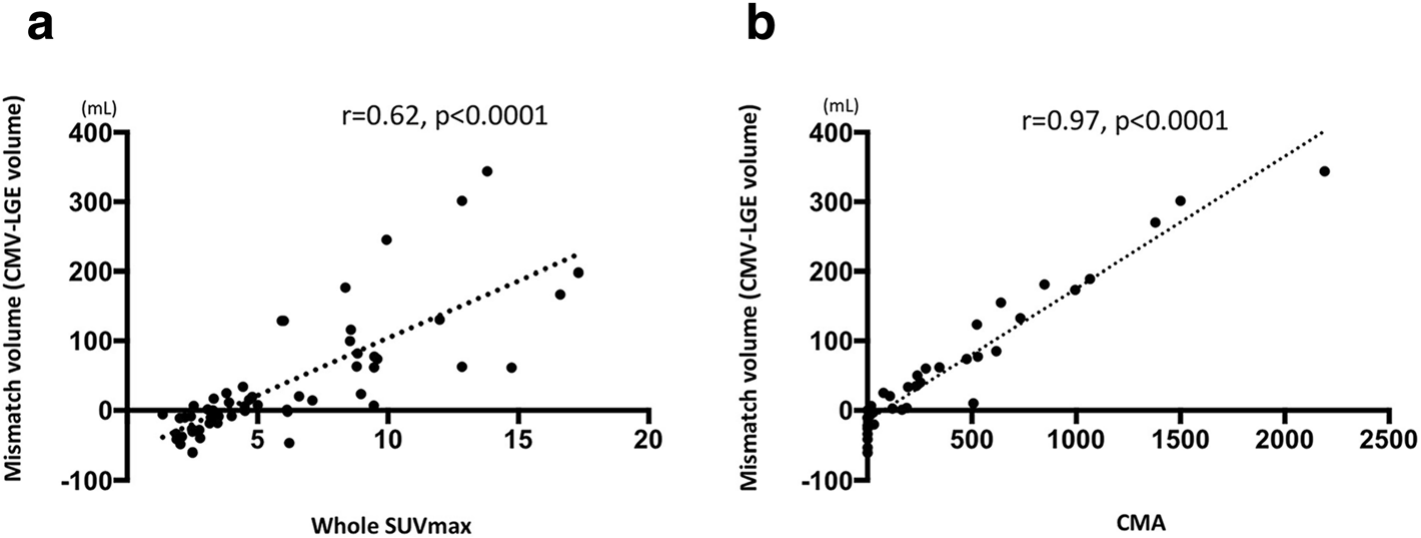
**a** Plot for %LV LGE and whole SUVmax and **b** for whole SUVmax and LVEF. Neither showed statistical significant correlation. **a**, **b** Association of the discrepancy of LGE volume and cardiac metabolic volume (CMV, volume of mean SUV over 2.7), and cardiac metabolic activity (CMA, multiplying CMV and mean SUV). Mismatch in LGE volume and CMV showed significant positive correlation to whole myocardial SUVmax and CMA (r = 0.62, *p* < 0.0001, r = 0.97, *p* < 0.0001, respectively)

The representative graphs are shown in Fig. 3. Significant negative correlation was found between LGE volume and LVEF (Fig. 3a). A weak but significant positive correlation was found between LVEF and whole SUVmax (Fig. 3b). Figure 4 shows the relationship between LGE volume and CMV. Mismatch in LGE volume and CMV showed a significant positive correlation to whole myocardial SUVmax (*r* = 0.62, *p* < 0.0001) (Fig. 4a). CMA also showed a significant correlation to mismatch volume (*r* = 0.62, *p* < 0.0001) (Fig. 4b).

### Analysis of segmental wall function, extent of LGE, and SUVmax by image fusion

**Fig. 5:**
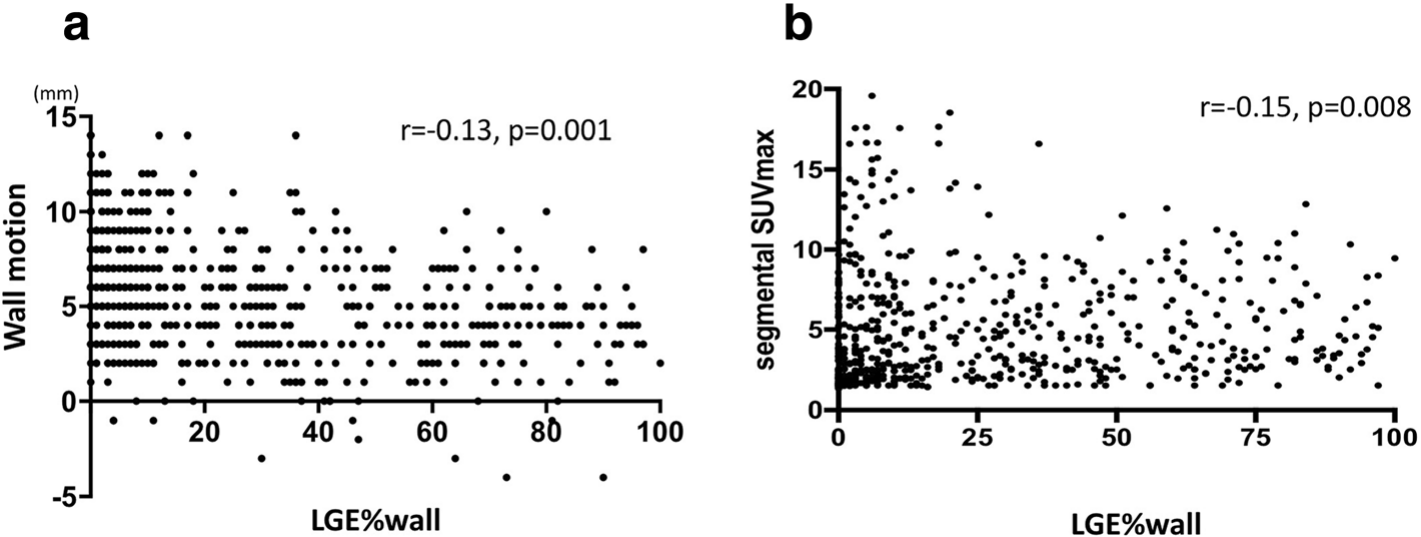
Segmental analysis of wall characteristics (LGE%wall vs. **a** segmental SUVmax, **b** wall motion) were shown in graphs. Significant inverse correlation was found between LGE%wall vs. segmental SUVmax and wall motion. LGE%wall and wall thickness did not show statistical significance. In the plots with a-dyskinetic wall motion, SUVmax was below 10. Wall thickness had significant correlation to segmental SUVmax, and thickened wall showed significant higher SUVmax

Segmental analysis for CMR, wall motion, and LGE%wall are shown in Fig. 5. Significant negative correlation was found between LGE%wall and wall motion (Spearman’s rank correlation coefficient *r* = − 0.13, *p* = 0.0011, Fig. 3a). Also, there was a significant negative correlation between LGE%wall and segmental SUVmax (Spearman’s rank correlation coefficient; *r* = 0.201, *p* < 0.0001, Fig. 3b).

### Case presentation

**Fig. 6:**
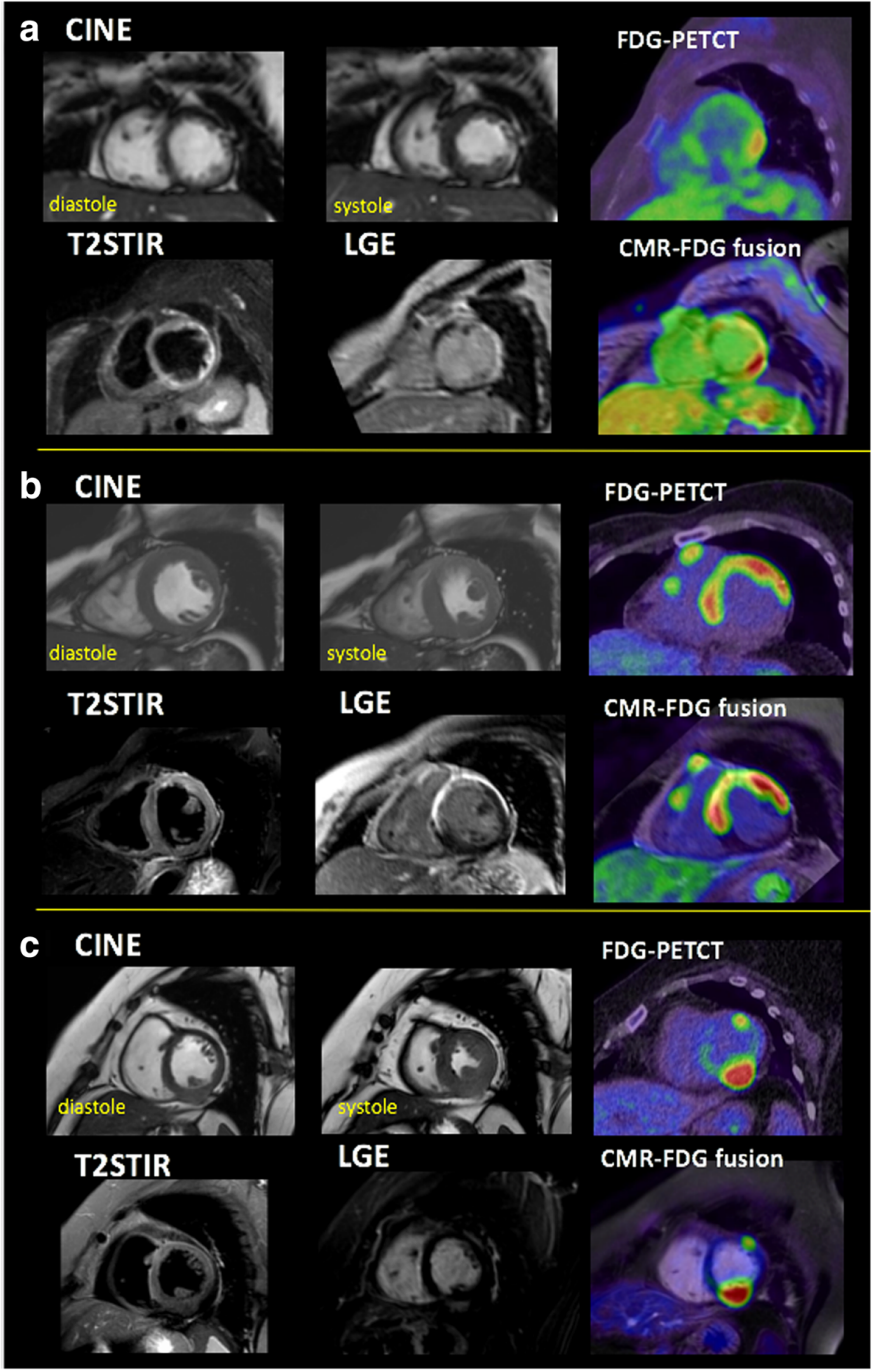
Three representative cases of CMR, FDG, and image fusion are shown. **a** 60-year-old female who had advanced heart failure with reduced cardiac function (EF = 30%) showed extensive LGE and dyskinetic-thinned wall in lateral. LGE volume, CMV, mismatch volume, and CMA were 45 mL, 10 mL, − 35.7 mL, and 32. FDG showed significant uptake in the area of positive LGE, and SUVmax value highest in the posterior edge of LGE site (segmental SUVmax = 6.2, LGE%wall = 54), while lower value of SUVmax = 3.4 was observed in dyskinetic lateral wall (LGE%wall = 96). **b** 54-year-old female with impaired cardiac function with sustained VT. CMR showed extensive LGE around basal area and significant FDG uptake (SUVmax = 9.6). LGE volume, CMV, mismatch volume, and CMA were 28.9 mL, 90.6 mL, 62 mL, and 345. Under fusion-guided analysis, basal antero-septal segment with high-grade fibrosis showed significant uptake (SUVmax 6.5). However, higher uptake (SUVmax = 9.5) was observed in basal septum and anterolateral wall which showed LGE%wall = 59. T2-weighted image showed high intensity in the antero-basal wall. **c** 66-year-old female who had systemic sarcoidosis. Her ejection fraction was slightly lower (LVEF = 47%). She was pointed out with premature ventricular contraction and driven for CMR. Short axis cine showed almost normal wall motion and volume in LV. However, posterior wall was remarkably thickened and with mild hyper-enhancement in LGE image (LGE%wall = 10%). In CMR-FDG fusion, significant focal FDG uptake (SUVmax = 16.6) was observed. T2 weighted image showed no abnormality. LGE volume was remarkably smaller than CMV (LGE volume, CMV, mismatch volume, and CMA were 14.6 mL, 155.4 mL, 170 mL, and 639)

Typical representative cases of CS in Fig. 6 showed typical cases for representing the relationship of LGE and corresponding FDG uptake. Case A had advanced heart failure with reduced EF which showed dyskinetic-thinned wall in lateral. Segmental SUVmax showed the highest value in the posterior edge with moderate LGE (LGE%wall = 54) and was 6.4. Anterolateral wall showed transmural LGE with lower FDG uptake (segmental SUVmax = 3.1–3.4). B presented with impaired cardiac function and sustained ventricular tachycardia. CMR showed extensive LGE around basal area and significant FDG uptake. Under image fusion, high FDG uptake was found in mid-antero-septal and anterolateral segment (LGE%wall = 59 and 56; segmental SUVmax = 9.2 and 9.3), and the segment with transmural LGE showed lower uptake (segmental SUVmax = 6.5). C had systemic sarcoidosis with only asymptomatic premature ventricular contraction. Cine-MRI showed mildly reduced EF (47%) with mild fibrosis in postero-lateral wall. However, remarkable focal thickened wall with significant high FDG uptake (segmental SUVmax = 16.6) was observed.

## Discussion

In this study, we investigated the relationship of the severity of myocardial fibrosis and the active inflammation in CS per patient and segment basis by quantitative methods. As is well known, definitive diagnosis of CS is quite challenging. Thus, we enrolled the patients with a focus on positive findings for both CMR and FDG as the criteria of clinical diagnosis (Hiraga et al. 2007; Yokoyama et al. 2015). In clinical routine, it was presumed that the image fusion might be quite challenging due to focal myocardial uptake in CS. We employed the technique to fuse CMR and ACCT automatically as a guide to succeed image fusion of spotty and blur FDG uptake. This technique enabled us to perform image fusion more accurately than conventional methods.

We performed quantitative LGE volume measurement using thresholds of over 6SD from null myocardium which enabled us to discriminate significant fibrosis from artifacts or noise. We also evaluated myocardial inflammation employing certain threshold (SUVmax > 2.7) according to previous reports (Osborne et al. 2014; Ahmadian et al. 2014). Several investigations had already reported FDG uptake varied despite of significant ventricular fibrosis. However, these investigations defined active inflammation by visual finding as “abnormal FDG uptake” without calculating SUV. Vita et al. suggested PSL therapy should be given to the subjects with abnormal FDG uptake even if CMR showed typical abnormal LGE (Vita et al. 2018). Osborn et al. and Amadian et al. firstly reported the usefulness of using SUVmax 2.7 and 4.1 (Osborne et al. 2014; Ahmadian et al. 2014). Especially, Osborn et al. emphasized threshold of SUV 2.7 seemed more useful when following up for the status post anti-inflammatory therapy. In this study, we focused on the relationship of activity of inflammation and the magnitude of fibrosis.

It should be noticed that all of the patients showed significant LGE consistent with CS because of the inclusion criteria. However, almost half of the patients who showed low FDG uptake (whole SUVmax < 2.7) were excluded. Vita et al. investigated 107 patients with CS and found 66% showed abnormal FDG uptake among those with positive LGE (Vita et al. 2018). Namely, 34% showed negative FDG uptake. This indicates that the discrepancy of CMR and FDG can be frequently seen. Our result also showed the extent of myocardial fibrosis was related to the cardiac dysfunction and the severity of remodeling in patients with positive for both LGE and FDG. This was a similar finding to previous reports (Ichinose et al. 2008; Matoh et al. 2008). Notably, the activity of myocardial inflammation showed a wide range, it reduced in patients with extensive fibrosis, and there was a discrepancy in the volume between myocardial fibrosis and inflammation among the enrolled patients. This discrepancy is basically due to fundamental differences of CMR and FDG. However, the discrepancy significantly associated with the activity of myocardial inflammation. This can be one of the explanations of negative FDG uptake among those with significant LGE.

Segmental SUVmax also showed regional variation in the wall analysis. The value ranged up to around 20 as the maximum in the myocardium with minimum to mild fibrosis, and the range converged down to around 10 with advanced fibrosis. And in the analysis of wall thickness, segmental FDG uptake correlated to wall thickness and significantly reduced in myocardium where remodeled thinned wall were present. In general, FDG shows significantly high uptake in granulomatous inflammation (Sebro et al. 2013). It is indicated that active inflammation had already reached its peak while the wall motion was preserved, and the inflammation may become no longer active when myocardium show advanced fibrosis and severely dyskinetic-thinned wall. This can be one of the potential reasons why invasive endo-myocardial biopsy may frequently fail to prove non-necronizing granuloma (Uemura et al. 1999). Ise et al. reported that corticosteroid therapy failed in patients with certain extent of myocardial fibrosis (LGE in LV over 20%). Our results can explain their report from the relationship of less active inflammation and advanced fibrosis which can be corticosteroid-refractory (Ise et al. 2014). The myocardium where the inflammation has burned out has turned into a scar for ventricular arrhythmia and heart failure, which are not treatable by anti-inflammatory therapy.

As shown in Fig. 5a, some segments showed active inflammation as segmental SUVmax around 15–20 with relatively preserved wall kinesis. Two case reports had presented the active cardiac sarcoidosis with preserved ejection fraction which showed remarkable high FDG uptake in myocardium (Radulescu et al. 2010; White et al. 2013). According to the results, the active inflammation may already manifest while myocardial damage is not yet overt. It should be proposed that therapeutic effects by the anti-inflammatory therapy are most beneficial on CS with early and active phase and can prevent disease progression before it turns to be dilated myocardium with scar of chronic inflammation. Further studies need to be proposed to identify early-staged and subclinical status CS by noninvasive approaches.

The former Japanese guideline in 1993 described thinned, aneurysmal, or/and thickened myocardium determined by echocardiography as one of the minor criteria. This conflicting criterion can be explained by the results of our study. The results showed significant thickened myocardial wall that maybe swollen due to the active inflammation, and the advanced fibrosis with less active inflammation showed thinned and aneurysmal wall. It is presumed that the former guideline has fragmentally captured the wall characteristic in the disease progression. The results of our study may contribute to the additional insights for re-classifying the pathological stage of CS to identify an appropriate candidate for anti-inflammatory therapy. Combined assessment by these imaging approaches is considered a useful framework for evaluation of CS, and fusion analysis enables us to comprehend the pathological status of the disease.

### Study limitation

Our study has several limitations. First, we defined significant myocardial FDG uptake as SUV 2.7 which may include physiological uptake, and it is difficult to affirm that those uptakes were completely excluded (Osborne et al. 2014). However, recent reports showed success for reducing normal myocardial glucose uptake by introducing a high-fat low-carbohydrate diet protocol. Second, SUVmax is affected by various factors (i.e., body weight, time from injection). However, considerable scientific contribution has been done using SUVmax as threshold in cancer field. Third, we excluded end or intra-myocardial dominant LGE which is needed to be differentiated from myocardial infarction or other cardiomyopathy. Even some cases were difficult to differ from ischemic cardiomyopathy, utmost effort was done to enroll CS by excluding significant coronary artery disease. Fourth, misalignment might occur either automatic or manual registration in image fusion.

## Conclusion

Comparative analysis of myocardial fibrosis and inflammation using CMR and FDG is a useful approach to access the pathophysiological stage in CS.

## Abbreviations

AHA: American Heart Association
CMA: Cardiac metabolic activity
CMR: Cardiovascular magnetic resonance imaging
CMV: Cardiac metabolic volume
CS: Cardiac sarcoidosis
FDG: ^18^F-fludeoxyglucose
LGE: Late gadolinium enhancement
SUV: Standardized uptake value
